# Hormesis: A Conversation with a Critic

**DOI:** 10.1289/ehp.0901002

**Published:** 2009-06-09

**Authors:** Edward J. Calabrese

**Affiliations:** Department of Public Health, Environmental Health Sciences Division, University of Massachusetts, Amherst, Massachusetts, USA

**Keywords:** adaptive response, biphasic, dose response, hormesis, hormetic, linear dose response, risk assessment, threshold dose response, U-shaped

## Abstract

**Objective:**

In this commentary I respond to points raised in the commentary by Mushak [Ad hoc and fast forward: the science and control of hormesis growth and development. Environ Health Perspect 117:1333–1338 (2009)], which principally concerns studies by me and my colleagues concerning the frequency of hormesis in toxicology.

**Discussion:**

In this commentary I demonstrate that Mushak’s analysis contains critical statistical errors and misunderstandings of statistical concepts that invalidate its conclusions concerning the frequency of hormesis in the toxicologic literature.

**Conclusions:**

In his commentary Mushak offers no significant new conceptual insights, and his key technical criticisms of hormesis frequency findings are unfounded.

The concept of hormesis has generated considerable interest within the biomedical and toxicologic communities over the past decade (Calabrese 2004, 2005, 2008c, 2009a, 2009b; Calabrese and Baldwin 2003b; Cook and Calabrese 2006a, 2006b; Hoffmann 2009; Mattson and Calabrese 2008; Scott 2008). It is within this context that Mushak (2009) critiques the growth and development of the hormesis concept within the scientific community, some of the past publications of its proponents, especially those dealing with frequency within the toxicologic literature, its generality, and the evolving definitional concept of hormesis. In this commentary I welcome the opportunity to address and rebut, where appropriate, these concerns. In general, Mushak presents a broad array of comments, some of which are technical, whereas others are in the realm of speculation and opinion. Mushak has raised a number of issues concerning the concept of hormesis, and I address these issues point by point below.

## The Frequency of the Hormetic (U-Shaped) Dose–Response Curve

Much of Mushak’s commentary (Mushak 2009) focuses on interpretation of results from Calabrese and Baldwin (2001), which reported on the frequency of hormetic (U-shaped) dose responses in the toxicologic literature. In that paper, we developed a database of dose–response curves obtained from the toxicologic literature and used three evaluative criteria to determine the presence of hormesis: *a*) statistical significance—at least one response below the no observed adverse effect level (NOAEL) that exhibits a statistically significant difference from the control group; *b*) data distribution—no 2 × SD/SEM overlap of at least one treatment response below the NOAEL with control response; and *c*) alternative quantitative—at least three doses below the NOAEL with responses ≥ 10% of control response. Using studies that met specific entry criteria, we (Calabrese and Baldwin 2001) reported that 37% (rounded up from 36.6%) of the dose responses satisfied one or more of these evaluative criteria.

In his critique of our study (Calabrese and Baldwin 2001), Mushak dismisses two of the three evaluative criteria and reduces the estimate of hormesis frequency from 37% to 11%. Mushak justifies this by indicating that only the first criterion—statistical significance—is an acceptable criterion for evaluating the presence of hormesis. To arrive at the 11% frequency value, Mushak reduced the number of dose responses that met at least one of the three evaluative criteria (245) to only dose responses that met the criterion for statistical significance (74) and divided by the total number of dose responses satisfying entry criteria (668). It is not appropriate to use only dose responses that satisfied the evaluative criteria for statistical significance in the numerator while retaining all possible dose responses for the denominator, as was done by Mushak (2009). Dose responses that could not meet the evaluation criterion for statistical significance (i.e., dose responses that did not have hypothesis testing) should not be included in the denominator in making the final frequency calculation. This point was explicitly documented in Table 1 of Calabrese and Baldwin (2001). If statistical significance was the only evaluative criterion, then there were 213 dose responses eligible, and 74 of these satisfied the statistical significance evaluation criteria. This is a 34.7% frequency, which is consistent with the 36.6% value we reported using all evaluative criteria (Calabrese and Baldwin 2001).

The argument to exclude the data distribution criterion from the calculation of hormesis frequency is overly restrictive. The use of nonoverlapping 95% confidence intervals (CIs) is generally recognized as an alternative approach to statistically distinguishing two means, similar to the hypothesis-testing approach where statistical significance is assessed by a *p*-value < α (used in the CI). In fact, considerable research is available within the statistical literature comparing hypothesis testing and 95% CIs. Cumming (2009) stated that

When 95% CIs on independent means do not overlap, the two tailed *p*-value is less than 0.05 and there is a statistically significant difference between the means. However, *p* for non-overlapping 95% CI is actually considerably smaller than 0.05: If the two CIs just touch, *p* is about 0.01 and the interval can overlap by as much as about half the length of one CI arm before *p* becomes as large as 0.05.

This perspective for 95% CIs for SDs has been repeatedly affirmed and emphasized in the statistical literature (Belia et al. 2005; Cumming and Finch 2005; Finch et al. 2002). These authors also developed a similar assessment when SEMs are employed. This analysis supports the use of nonoverlapping CIs as an evaluative criterion for evaluating the frequency of hormesis.

With regard to the third evaluative criterion—alternative quantitative—we (Calabrese and Baldwin 2001) reported that it was twice as difficult to satisfy this criterion than the other two. Specifically, there were 75 dose responses meeting the statistical significance and data distribution criteria that also had three or more responses below the NOAEL. This observation permitted the opportunity to assess what proportion of these 75 dose responses would have also satisfied the alternative quantitative criterion, which provides a means of judging which criteria were more stringent. Of the 75 dose responses, only 38 (50.6%) would have satisfied the alternative quantitative criterion, suggesting that this criterion is about twice as rigorous as the other two criteria. This interpretation indicates that the alternative quantitative criterion is reasonably conservative. Excluding this criterion from the calculation of hormesis frequency is not warranted. Our calculations indicate that if the dose responses meeting the alternative quantitative criterion were normalized with the same rigor as the other two, the frequency of hormesis would exceed the 37% value reported previously (Calabrese and Baldwin 2001). Conversely, even if dose responses meeting this criterion were excluded and the denominator adjusted accordingly, hormesis frequency would remain essentially unchanged.

Experts may not agree with the different criteria in the evaluation of dose responses to determine frequency of hormesis. Exclusion of any of the evaluation criteria, however, must be based on sound scientific and statistical principles. Most important, it is essential to use the appropriate denominator in any calculation of a frequency of occurrence. Not doing so after eliminating an evaluation criterion is an error made by Mushak (2009). If one excludes the cases to which Mushak objects and calculates a frequency using the correct number of possible cases in the denominator, the estimate of hormesis frequency is very similar to that reported we reported (Calabrese and Baldwin 2001).

Mushak (2009) also fails to point out additional means by which the methodology employed to estimate frequency was conservative and likely led to further underestimates of the actual hormetic frequency. In another study (Calabrese and Baldwin 2003b), we presented evidence that responses immediately below the estimated threshold (i.e., NOAEL) gave evidence of modest toxicity. This is most likely because the NOAEL may commonly express a limited degree of toxicity even though it does not achieve statistical significance. We refer to this as “residual” toxicity. The key point is that the occurrence of residual toxicity for the first response below the NOAEL biases against observing possible hormetic responses.

## Concerns about the Published Record

Mushak (2009) also raises several concerns related to previously published work concerning the hormesis hypothesis. For example, we (Calabrese and Baldwin 2001) made an error in Table 5 in tabulating the number of dose responses to estimate a false-positive hormesis response rate. Either an addition error or a typing error reversed a “7” and a “5” (i.e., 57 versus 75). This error led us to calculate a 3.8% positive error rate when it should have been 5.2%. This correction does not affect the conclusions of the paper.

Mushak also points out that we (Calabrese and Baldwin 2001) listed 1,089 data points below the NOAEL, whereas 1,791 were listed in another paper (Calabrese and Baldwin 2003b). Simple methodologic differences account for the disparate numbers. We (Calabrese and Baldwin 2001) reported 1,089 data points based on using two criteria, statistical significance and data distribution, which was appropriate for the specific analysis in Table 4 of that paper for estimation of false positives. In another paper (Calabrese and Baldwin 2003a), dose responses based on all three evaluation criteria were combined to obtain a larger number. This approach was appropriate to the conditions studied in that particular paper.

Mushak (2009) has additional concerns about the possibility that some dose–response data in support of the hormesis hypothesis may have been published more than once because of a convergence of publications in certain years and therefore possibly double entered into the hormesis database (Calabrese and Baldwin 2001). We have conducted a detailed assessment of all references used in the hormesis frequency database and found no evidence to support this possibility. Approximately 95% of articles were from research teams with only one publication entry into the database. For those research teams with more than one publication entry into the database, none had duplicate data entries. Double entries cannot explain the relatively high frequency of hormesis in the database we reported (Calabrese and Baldwin 2001).

Finally, Mushak calls for more transparency in the presentation of our papers, especially Calabrese and Baldwin (2001). This critique is puzzling given the detailed description of the methods, the description of the entry and evaluative criteria, and the presentation and interpretation of the results. Furthermore, researchers may apply our methodology to any data set for comparison.

## Validation of the Model

Mushak (2009) argues that the model employed in studies on hormesis needs to be validated and tested for sensitivity and specificity. Our multiple evaluative methods were designed to validate the general predictive capacity of the threshold and hormetic dose–response models. In the case of our 2001 study (Calabrese and Baldwin 2001), the approach we used was supported with the data provided on specificity [e.g., false positive (type 1 error)] and sensitivity [e.g., false negative (type 2 error)]. The hormesis frequency estimate was corrected for false-positive and false-negative values. These findings indicated that the hormesis frequency estimate was not particularly susceptible to false-positive error.

## Poor Predictability of the Threshold Model

In our 2003 study (Calabrese and Baldwin 2003b) we concluded that hormesis was more common than the threshold dose response based on the observation that approximately 1,800 responses below the estimated threshold were nonrandomly distributed about the control in a manner to strongly support an hormetic interpretation. The ratio of above to below (and equal to) control values was 2.5 to 1. Mushak (2009), however, argues that “data points were not gathered from a purely random sampling within the main database . . . .” A careful reading of Calabrese and Baldwin (2003a) would find that the nearly 1,800 responses constituted the entire database of the dose responses satisfying the entry criteria. It does not seem logical to criticize a sample as nonrepresentative when it is the entire database. If, on the other hand, all 20,285 screened articles were used in determining the frequency of hormesis, it would introduce substantial negative bias. Clearly, many of the sampled studies were not designed to examine hormesis, and others did not even measure a biological response to a chemical agent. *A priori* entry criteria were used to determine the suitability of a data set for inclusion in the analysis of hormesis frequency.

## Occurrence of False Positives

Mushak (2009) questions whether the occurrence of false-positive values in the Calabrese and Baldwin (2001) paper were highest with the statistical significance criterion, lower with the data distribution, and lowest with the alternative quantitative criteria (three responses ≥ 110% of controls). He concluded that the “least problematic and the most problematic approaches show the highest and lowest false-positive rates, respectively.” We (Calabrese and Baldwin 2001) indicated that the alternative quantitative approach appears to be twice as rigorous as statistical significance and data distribution criteria. Thus, it is likely that there would be lower false-positive rates for methods that are twice as rigorous as the statistical significance/data distribution criteria, and this is what we observed (see Table 5 of Calabrese and Baldwin 2001).

## Generality of the Phenomenon

Mushak (2009) also claims that hormesis may not be highly generalized. The findings shown in Table 3 of Calabrese and Baldwin (2001), as well as Calabrese and Blain (2005), demonstrate that hormetic responses satisfying the evaluative criteria are widely distributed across biological systems, ranging from plants to microbes to invertebrates and vertebrates. This observation is extremely commonplace in numerous other publications. Furthermore, hormetic findings have been reported for large numbers of agents and are independent of chemical class. The hormetic response is also independent of the end point measured. These observations indicate that the principle of hormesis can be generalized widely. This does not mean, however, that the hormetic response will occur in all cases. In fact, we have identified experimental conditions in which hormesis will not be expected, such as with a very low background disease incidence. Others have reported that there are specific experimental conditions that favor or minimize the manifestation of hormetic responses (Vichi and Tritton 1992). Such restrictions, however, do not alter the conclusion that hormesis appears to occur over a wide range of biological conditions and models.

In addition, Mushak is also concerned about the generality of our findings obtained from all the articles published in three journals typically publishing papers from environmental toxicology and pharmacology/biomedical sciences. Although it may be of interest to extend our work to other journals that focus on other or similar end points, our findings revealed that there were essentially no major differences among the journals with respect to the frequency of hormesis, regardless of the evaluative criteria employed. These find-ings were consistent with several thousand articles in the peer-reviewed literature showing hormesis across model, end point, and chemical class, making a strong argument that the hormesis concept is a very general one.

## Questions Concerning Statistical Significance

Mushak (2009) raises the issue that the hormesis data used in the frequency evaluation (Calabrese and Baldwin 2001) should be corrected for multiple tests of statistical analysis on the same data, which could falsely give the appearance of significance, as 1 of every 20 hypothesis tests may be expected to be significant at the α = 0.05 level purely due to chance. Furthermore, the probability of obtaining a statistically significant result with *n* tests at this level of significance is 1 − 0.95*n*(1 − probability of not getting a significant result with *n* tests). The key phrase is “on the same data.” Such corrections may be applied when multiple comparisons are tested in the same experimental system for the same end points and have the same likelihood for false-positive findings. In the instance of the hormetic database, this is not the case, as the data are obtained from highly diverse experimental systems and instruments and different biological models, and using different end points, study designs, and other factors. Standard statistical correction methods, along with their basic assumptions, were not designed to address this issue. Of potential relevance may be the area of meta-analysis in the field of epidemiology in which different study findings are compared in an integrative manner. However, even in this case there is considerably greater homogeneity of end point and method than occurs with respect to the hormesis database that is not restricted by biological model, end point, chemical class, and experimental methods. Furthermore, in a major proportion of the studies showing hormesis, the findings are not the result of a single assay performed only once. It is typical for investigators to replicate their results via various approaches prior to publishing their findings. We typically follow the research of individual investigators over numerous publications to trace and confirm the occurrence and consistency of the hormetic response. We also obtain dissertations by new investigators as follow-up to their journal publications on hormesis to obtain more evidence of the consistency of the find-ings. Investigators commonly are publishing data that are highly reproducible and often representative of numerous other experiments in their laboratories. These studies typically lead to deeper mechanistic insight, generalized to other biological models, often with highly consistent results. Although it is likely that there is some proportion of false-positive values in the hormesis database used to construct the frequency estimates, discussions with biostatisticians and epidemiologists indicate that current correction methods are not readily applicable to the hormesis database, suggesting the need for further research. The suggestion that a correction factor for possible false-positive findings should be applied may be a useful concept, but the critique as it relates to the special demands of the hormetic database is not appropriate.

## The National Cancer Institute Yeast Data Set

In his commentary, Mushak (2009) raises concerns about our studies concerning the National Cancer Institute (NCI) yeast data set (Calabrese et al. 2006a, 2006b, 2008), based largely on a letter to the editor by Crump (2007). We maintain that information contained in a letter to the editor is not peer reviewed and therefore lacks normal standards to ensure credibility. More important, Crump reanalyzed the data in a manner that was not reported by the original investigators and was specifically refuted by them (Calabrese et al. 2006b). His approach also introduced 8-fold more variability into the analysis. This extra variability resulted in the findings supportive of hormesis failing to achieve statistical significance. Crump justified his actions because the original data of the NCI were lost, and he concluded that the way he analyzed the data was as likely as what the original investigators claimed. I (Calabrese et al. 2007) responded to this claim by indicating that we had interviewed the NCI investigators prior to starting our work. The NCI research group was clear, firm, and consistent in their descriptions of their research and statistical methods. After Crump’s letter was published, the NCI group was reinterviewed and provided unequivocal confirmation verbally and in writing of their prior statements. In my opinion, Crump (2007) was incorrect in his assumptions concerning the reanalysis of the data. Furthermore, we surveyed a large number of biomedical scientists who perform similar assessments as conducted by the NCI team. Essentially all performed their statistical analyses as reported by the NCI team. No group or individual performed statistical analyses as Crump did. A similar survey of biostatisticians at leading research centers indicated that no one supported Crump’s approach, whereas the methodology of the NCI was consistently affirmed. Finally, a survey of the peer-reviewed literature of publications with 96-well plate assays indicated that none used Crump’s method, whereas most used the procedure of the NCI. The National Institutes of Health also requires that original data be held for 3 years after the end of the grant. Thus, a significant fraction of what we know in science is based on published results for which the original data may not be available. In sum, it is not logical to claim, as Mushak (2009) does, that Crump’s method was as plausible as that reported by the NCI researchers. Beyond his reliance on the discredited analysis of Crump (2007), Mushak offers no technical criticisms of our yeast frequency studies (Calabrese et al. 2006a, 2006b, 2008). In fact, the NCI data set has consistently revealed high hormetic dose–response frequency using multiple types of modeling methods, with very differing analytic strategies. Hormetic findings were common, robust, and rigorously determined.

## Data from the National Toxicology Program

Mushak (2009) also raises concerns about the frequency of hormesis reported in an analysis of data obtained from the National Toxicology Program (NTP) (Calabrese and Baldwin 2003a). We asessed 59 environmentally relevant chemicals in the NTP toxicity database for their capacity to exhibit hormesis in dose–response curves for growth, as evidenced by assessment of weight gain. The NTP study included bioassays involving both mice and rats. We (Calabrese and Baldwin 2003a) reported that for the 409 dose responses reported, there was evidence of hormesis in 128 (31%) cases. In that study we used a well-described six-point rating scale to define the strength of the hormesis response: no to low, low, low to moderate, moderate, moderate to high, and high. The 31% value was obtained by adding all of the dose responses that showed low, low-to-moderate, moderate, moderate-to-high, and high evidence of hormesis. It is true that the large majority of the 128 dose responses showing hormesis were classified as low evidence (*n =* 98). The fact that a large number of dose responses in the NTP data set had a low evidence rating does not detract from the fact that a hormesis response was detected using the rating scale. Moreover, the data set obtained from the NTP included dose-ranging studies, usually including five doses and a control for 2-week and 13-week exposure periods. These studies typically use higher doses and are not designed to detect effects at doses at or below the NOAEL. Nonetheless, hormetic responses were still quite common.

In our 2005 paper (Calabrese and Blain 2005) we specifically compared the rigor of the evaluative criteria of the hormesis frequency database (Calabrese and Baldwin 2001) with that of the larger and more general hormesis database. This is relevant to the NTP assessment, because the NTP data were evaluated using the criteria of the general hormesis database. Even though these databases were designed for different purposes and employed different evaluative criteria, when all 245 dose responses that satisfied the evaluative criteria (i.e., hormesis designation) in the hormesis frequency database (Calabrese and Baldwin 2001) were assessed using the scoring system employed in the general hormesis database (Calabrese and Baldwin 2003a), the distribution of ranked scores was very similar for both databases (Table 1). That is, those dose responses satisfying evaluative criteria in the frequency database showed the same quantitative distribution patterns for low, moderate, and high evidence of hormesis as is seen in the general hormesis database. These findings, therefore, revealed a high level of agreement between the two different but complementary evaluative methodologies. This strongly suggests that both methodologies had a comparable level of evaluative rigor. Thus, Mushak’s suggestion that dose responses in the low evidence category of the NTP assessment should be excluded (Mushak 2009) is not supported by the data.

## Language

Mushak (2009) expresses concern about how hormesis has been defined and the evolution of the conceptual history of hormesis. First, because science is dynamic, progressive insights will always yield refinements of understanding. This is the nature of scientific inquiry, and it is inherently self-correcting. One only has to look at the changing conceptualization of evolution since it was postulated first by Charles Darwin. Second, since the paper “Defining Hormesis” (Calabrese and Baldwin 2002), many biomedical scientists have used the hormesis concept to help explain their own findings. The growing number of scientists has brought forth new levels of biological organization (i.e., molecular, cellular, organismal, and ecologic) in which hormesis is studied, leading to new and evolving perspectives.

## Ad hoc Retrospective Assessment

Retrospective and integrative assessments of published literature can be very important in the process of scientific discovery. The retrospective assessment approach has not been presented as better than a purely experimental prospective hypothesis testing method but complementary to it. In fact, narrow experimental studies would not have addressed the specific issue of frequency in as meaningful a way. The ad hoc approach has provided a valuable foundation to explore the hormesis concept within a broader and more integrative fashion. It has revealed that the hormesis phenomenon has been reported in multiple fields of biology concerned with dose-related phenomena, by many hundreds of independent research teams, and has passed numerous and independent peer reviews. This supports the presence of a general biological principle. That it may not be detected under certain circumstances should not be surprising.

In addition, the ad hoc perspective has been applied to large amounts of data in which hormesis was not appreciated by the original investigators. It has likewise been applied to studies where it was built into the original study hypothesis. Retrospective methods such as meta-analysis in epidemiology are now viewed as mainstream, offering critical insights to that discipline. It is also standard in epidemiologic investigations to use secondary data sets for investigation, hypothesis generation, and testing. Numerous epidemiologic dissertations are based entirely on the ad hoc use of such secondary data sets. Furthermore, many outstanding research discoveries were the unintended offshoots of serendipitous observations even though the original experiments were not designed to study the phenomenon. In fact, every researcher who reflects on their data is acting in an ad hoc manner. This is called “following their data” rather than the idea that led to the data.

## The Achievements of Hormesis in the Last 15 Years

Mushak (2009) concludes that hormesis has generally had little impact over the last 15 years. This assertion simply fails to acknowledge the gains that hormesis has made within the larger scientific community. For example, the number of citations in the scientific literature on hormesis (or hormetic) has rapidly increased. In 2008 alone, the Web of Science lists nearly 2,300 citations. This is up from only 16/year throughout the 1980s. This is an indication that many researchers are studying and observing hormesis, using multiple biological systems, following a broad range of hypotheses, with wide and varied funding sources, and that their research has passed numerous independent peer reviews. All leading (and nonleading) toxicologic textbooks contain sections on hormesis, giving it clear standing in the field. Also not acknowledged is the fact that the concept of hormesis is central to a range of biomedical areas such as with anxiolytic drugs (Calabrese 2008b), antiseizure drugs (Calabrese 2008d), memory drugs (Calabrese 2008a), and others (Calabrese 2008c). In fact, all drugs approved by the Food and Drug Administration for Alzheimer’s disease follow the hormetic dose response (Calabrese 2008a). Hormesis is now a major influence in aging research (Mattson and Calabrese 2008; Rattan 2008) as well as in exercise science (Radak et al. 2008) and plant biology/weed science (Belz 2008; Calabrese and Blain 2009), among others. The French Academy of Sciences/National Academy of Medicine acknowledged support of the hormesis concept in 2005 (Academie Nationale de Medecine 2005). A key feature is that most research supportive of hormesis has been performed totally independently of me and my colleagues, including its translation from animal studies into the clinic and human populations.

Mushak also states that hormesis has not been adopted by public agencies for inclusion in health and regulatory policies probably because of the singular nature of hormesis research and directions followed in hormesis methodologies. This interpretation is actually incorrect, as noted above for entire areas of pharmaceuticals. With respect to health and regulatory policies, this comment is highly speculative. It is just as likely that hormesis has not been included in risk assessment methodology and risk characterization by regulatory agencies because these agencies are highly conservative. A change in policy concerning how to interpret biological activity at or near the threshold will require considerable consensus between scientists and policy specialists and must address multiple political issues, cost–benefit analyses, and concerns of various advocacy groups and the public. A change in policy to accommodate the principle of hormesis may also raise issues relative to past regulatory actions and records of decisions for environmental cleanups. In any case, the perspective that hormesis has had little impact over the last 15 years is inaccurate and unbalanced, presenting a reader with a distortion of the progress, accomplishments, and concept penetration of hormesis within the scientific community.

## Conclusions

Hormesis has become widely accepted within the biomedical and toxicologic communities. The concept in now included in leading textbooks in toxicology and continues to be cited at a rapidly increasing rate in the scientific literature. Research supports the generality of the principle in numerous plant, microbial, invertebrate, and vertebrate models, including humans, while being independent of the end point measured and chemical class assessed. The hormetic dose response has also outperformed standard default dose–response models used by regulatory agencies (i.e., threshold and linear at low doses) in direct comparisons in making accurate predictions of responses below estimated toxicologic and pharmacologic thresholds. In his commentary Mushak (2009) raises questions about the frequency of hormesis, its definition, and how it may be studied. Based on his analysis, hormesis has not made a substantial contribution to the field and, as a concept, is now only less negligible than it was 15 years ago. In my opinion, his commentary offers no significant conceptual insights concerning hormesis, and its key technical criticisms of studies concerning the occurrence of hormesis are seriously flawed. Erroneous conclusions concerning hormesis are based primarily on unsubstantiated dismissal of key evaluative criteria to assess the frequency of hormesis, miscalculation of the remaining data leading to an inappropriate frequency estimate, and reliance on scientifically unproven analytical approaches of key supportive data sets. It is unfortunate that Mushak does not recognize the broad acceptance and utilization of the hormesis concept over the last 15 years.

## Figures and Tables

**Table 1:** Comparison of the scores for the dose responses in the hormesis frequency database with the general hormesis database.

	Frequency database^a^ [no. (%)]	Hormesis database^b^ [no. (%)]
Total	245 (100)	5,632 (100)
Performed hypothesis testing	87 (36)	2,309 (41)
Low	130 (53)	3,185 (57)
Low–moderate	65 (27)	1,040 (19)
Moderate	28 (11)	566 (10)
Moderate–high	12 (5)	250 (4)
High	10 (4)	551 (10)

a Data from Calabrese and Baldwin 2001.

b Data from Calabrese and Blain 2005.
